# Intra-rater reliability of the modified Tardieu scale to quantify spasticity in elbow flexors and ankle plantar flexors in adult stroke subjects

**DOI:** 10.4103/0972-2327.78045

**Published:** 2011

**Authors:** Priyanka Singh, Abraham M. Joshua, Sailakshmi Ganeshan, Sucharitha Suresh

**Affiliations:** Sikkim Manipal Institute of Medical Sciences, Gangtok, Sikkim, India; 1 Department of Physiotherapy, Kasturba Medical College, Mangalore, Karnataka, India; 2 Department of Community Medicine, Father Muller Medical College, Mangalore, Karnataka, India

**Keywords:** Reliability, spasticity, stroke, Tardieu scale

## Abstract

**Objectives:**

The purpose of this study was to investigate Iintra-rater reliability of the Modified Tardieu Scale (MTS) in elbow flexors and ankle plantar flexors in adult subjects with stroke.

**Materials and Methods:**

A total of 91 subjects with stroke participated in this test-retest study. Intra-rater reliability of the MTS was investigated by a qualified and trained physiotherapist for elbow flexors and ankle plantar flexors in two sessions. A rater was one who performed the procedure and an observer only records the angles so that the rater was blinded to findings. Outcome measures in this study were measurable components of MTS, which are angle of muscle reaction (R1), passive range of motion (R2), dynamic component (R2-R1), and quality of muscle reaction (grade 0 – 4) termed as MTS score.

**Results:**

Intra-rater reliability of MTS was very good for R1, R2, R2-R1, and MTS score (ICC > 0.85, *P*<0.0001) across two sessions in elbow flexors and ankle plantar flexors.

**Conclusion:**

MTS is a reliable clinical tool for measurement of spasticity in the elbow flexors and ankle plantar flexors in adult subjects with stroke.

## Introduction

Stroke is one of the major conditions which cause spasticity.[1] Prevalence of spasticity after stroke has been reported in 50% of subjects with stroke.[1,2] Spasticity is reported to develop within one month of an acute stroke.[3,4]

Spasticity is best described as ‘a motor disorder characterized by a velocity dependent increase in tonic stretch reflexes with exaggerated tendon jerks, resulting from hyper excitability of the stretch reflex.’[5] This definition suggests that the abnormality underlying spasticity is hyper excitability of the stretch reflex, both tonic and phasic components, which can result in an increased resistance to passive movement.[6] Measurement of spasticity can be done by clinical and laboratory methods. The currently used clinical measurement tools for spasticity are the Ashworth and Modified Ashworth scales (MAS),[7] but their validity has been questioned as they do not measure velocity-dependent aspect of spasticity.[8,9] Vattanasilp *et al*. also described Ashworth scale as a grading of muscle stiffness, which is unable to ‘differentiate’ between ‘the neural and peripheral contributions.’[10]

The neural component is due to overactive stretch reflex. The peripheral or biomechanical component is due to changes in soft tissue including tendons, ligaments, and the joints themselves resulting in decreased compliance.[11] The resistance felt is not solely due to the neural component but also due to biomechanical factors (such as soft tissue compliance and joint integrity).[12] Differentiation between the neural component and soft tissue changes is necessary as they respond to different types of treatment.[13]

In 1954, Tardieu *et al*. developed a clinical scale, known as Tardieu Scale, to measure the spasticity[14] which was further modified by Held and Peierrot-Deseilligny,[15] and was later modified by Boyd and Graham, presently known as Modified Tardieu Scale (MTS).[16]

The MTS determines the passive range of movement (PROM) at different movement velocities, with the relative difference between a slow and a fast velocity passive stretch determining the dynamic component of the muscle contracture.[17] With the MTS, two resulting joint angles are measured by goniometer which include the R1 angle which is the ‘angle of catch’ after a fast velocity stretch and the R2 angle defined as the passive joint range of movement following a slow velocity stretch.[11] The R2–R1 value indicates the level of dynamic component of spasticity in the muscle. A larger difference between R1 and R2 means large dynamic component, whereas a small difference between R1 and R2 means static contracture in the muscle.[16] The MTS uses standardized procedures to measure quality of muscle reaction at the following specified three velocities that can be applied to the muscle: as slow as possible (V1), the speed of the limb falling under gravity (V2), and as fast as possible (V3). V1 velocity is used to determine the R2 joint angle and either V2 or V3 is used to determine the angle of catch (R1) depending on the muscle to be tested. The ‘catch’ following a fast velocity stretch is hypothesized to be the result of an overactive stretch reflex present in spastic muscles.[16]

On the review of the published literature, we found that major studies have been done in children population. Boyd *et al*. demonstrated good intra-rater reliability for MTS in children with cerebral palsy (CP) on comparison of MTS with MAS.[18] In another study, Fosang *et al*. demonstrated variable intra-rater reliability and acceptable inter-rater reliability, although PROM measurements showed disparities.[19] A study done by Mackey *et al*.[20] showed large intersessional variability in Tardieu Scale measures of R2 and R1 as well as R2-R1 difference while studying measurement of biceps spasticity in 10 children with hemiplegic CP. Yam and Leung[21] demonstrated low inter-rater reliability for MTS and MAS in four muscle groups in lower limbs of children with spastic CP. Among the limited literature in adult population, Mehrholz *et al*. has demonstrated higher test-retest and poor to moderate inter-rater reliability for MTS as compared with MAS in a study done in adult severe brain injury subjects.[22] The inter-rater reliability of MTS for elbow flexor was moderately high in a study done by Ansari *et al*. in adult subjects with hemiplegia.[23] There is less published literature regarding reliability of MTS in adult subjects with stroke which prevents recommendation of this tool in this population. The purpose of this study was to investigate intra-rater reliability of the MTS in elbow flexors and ankle plantar flexors in large population of adult subjects with stroke.

## Materials and Methods

A test-retest design was adopted to conduct intra-rater reliability of MTS in adult subjects with stroke. Institutional ethics committee approved the study. This study recruited 130 subjects with stroke in two multispecialty K.M.C hospitals at Mangalore during time frame of this study. Of this, 39 subjects were excluded because 29 subjects refused to participate in the study, seven subjects were not able to understand instructions, and three subjects were medically unstable. A total of 91 subjects were selected for the study based on inclusion and exclusion criteria. Inclusion criteria were (1) medically stable acute stroke subjects at least after 1 month of onset, (2) age 45 to 85 years, (3) able to follow instructions for test procedure, and (4) MMSE score >24. Exclusion criteria were history of pain or surgery in the joint of interest, previous episode of transient ischemic attack, and patient on tone-modifying drug. All subjects provided their written informed consent.

### Procedure

Baseline characteristics were recorded for all the subjects as shown in Table 1. MTS was administered on the elbow flexors in the upper limb and ankle plantar flexors in the lower limb of the affected side. To test the intra-rater reliability, MTS was administered twice by rater on every subject at an interval of two days on same time of the day. Rater had received training under two Neuro-physiotherapists who had experience of stroke rehabilitation over 10 years in a multispecialty hospital. Rater had also attended a workshop of national level for three days. Rater took the readings with the help of an observer who was blinded to the study and rater herself was blinded to findings. All subjects were tested in the same position for both the tests. Subjects were instructed to completely relax for at least 10 minutes before all tests. Standardized resting limb positions were followed for elbow flexor and ankle plantar flexor measurements as described in a previous study.[16] Goniometer was used to measure range of motion and the equipment was rounded to 1 degree for accuracy. Placement of goniometer for measurement of angle of muscle reaction R1 and R2 was adapted.[24]

**Table 1:** Subjects baseline characteristics

Characteristics	Subjects (N = 91)
Age (years)	
Mean ± SD	64.0 ± 11.1
Range	45–80
Gender	
Men	54
Women	37
Type of stroke	
Ischemic	61
Hemorrhagic	30
Side of involvement	
Right	49
Left	42
Duration of illness days	
Mean ± SD	57.5 ± 13.5
Range	32–78
MMSE score	
Mean ± SD	27.3 ±·57
Range	27–29

SD, standard deviation; MMSE, Mini mental state examination

The test procedure was as follows: the subjects were made to sit on a chair with shoulder in adduction for elbow flexors. A universal goniometer was used for the test procedure. The lateral epicondyle of humerus was marked with a marker pen and a point was marked on the acromion process for reference. A line was drawn joining these two points (first line). The second line was drawn from the radial head to the radial styloid process, after positioning of the axis over the lateral epicondyle of humerus with stabilizing arm along the first line and movable arm along the second line. The goniometer was fixed by two Velcro (2 inch width) around the arm and forearm.

The joint was moved first with a very slow-stretching velocity (V1) from elbow flexion to extension to measure the PROM by counting as 1001, 1002, 1003……onwards. During this maneuver, the catch was noted by the rater and R2 was documented by the observer. Quality of muscle reaction (MTS scores) ranging from 0 to 4 grades were rated by the rater at the stretching velocity of V2. At last, the angle of muscle reaction was measured at the point of resistance to the fastest stretching velocity V2 by counting 1, 2, 3…..onwards. The angle of catch (R1) was noted by the observer. Throughout the procedure, the rater was blinded by covering the goniometer with opaque tape and observer documented all values. The test procedure was repeated by rater after two days interval to remeasure the R1, R2, and MTS scores parameters.

The test procedure for the ankle plantar flexors was as follows: all subjects were placed in supine position with ankle joint out of the couch with knee in extension. The rater who performed the test procedure marked the lateral aspect of lateral malleolus and another point was marked on the head of fibula for reference by a removable marker. The first line was drawn joining these two points and the second line was drawn parallel to the lateral aspect of the fifth metatarsal. After positioning of the axis over the lateral malleolus with stabilizing arm along the first line and movable arm along the second line, the goniometer was fixed by two Velcro over midfoot and above ankle. The rater moved the ankle joint first with a very slow-stretching velocity (V1) and then fast-stretching velocity (V3),[16] from plantar flexion towards dorsiflexion. Measurement of RI, R2, and scores of quality of muscle reaction was recorded as per the same procedure mentioned above for elbow flexors.

### Data analysis

Data was analyzed by SPSS (version 16.0, SPSS Inc, Chicago, IL). Data analysis was blinded. Intraclass correlation coefficient (ICC) was used as statistical measure to find out intra-rater reliability. ICC values were interpreted as follows: <0.2, poor agreement; 0.21 to 0.4, fair agreement; 0.41 to 0.6, moderate agreement; 0.61 to 0.8, good agreement; and 0.81 to 1.0, very good agreement.[25]

## Results

A total of 91 adult subjects with stroke were included in this study, of which 54 were men and 37 were women. Baseline characteristics of all subjects are given in Table 1.

Intra-rater agreement for elbow flexors for R1, R2, and R2-R1 showed very good agreement of 0.998, 0.978, and 0.991, respectively. MTS score showed ICC of 0.847. Intra-rater agreement for plantar flexors also has very good agreement for all the variables as shown in Table 2. ICC value for R1 was 0.990, R2 was 0.995, R2-R1 was 0.907, and MTS score was 0.863.

**Table 2:** Intra-rater agreement across two sessions (elbow flexors and ankle plantar flexors)

MTS measures	Sessions	Elbow flexor				Ankle plantar flexor			
		Mean (SD)	ICC	95% CI	*P* value	Mean (SD)	ICC	95% CI	*P* value
R1	1	124.2 (35.9)	·998	·997–999	<0.0001	-10.7 (6.4)	·990	·985–994	<0.0001
	2	124.1 (35.3)				-10.8 (6.6)			
R2	1	165.1 (10.6)	·978	·966–986	<0.0001	12.8 (10.0)	·995	·992–997	<0.0001
	2	165.8 (10·4)				12.5 (9.8)			
R2-R1	1	40.8 (30.6)	·991	·986–994	<0.0001	4.16 (8.3)	·907	·856–939	<0.0001
	2	41.6 (30.1)				3.13 (8.5)			
MTS scores	1	2.13 (40)	·847	·769–899	<0.0001	2.71 (0.45)	·863	·793–910	<0.0001
	2	2.17 (41)				2.63 (0.52)			

MTS, Modified Tardieu scale; SD, Standard deviation; SEM, Standard error of mean; ICC, Intraclass correlation coefficient

## Discussion

In this study, we are reporting intra-rater reliability of MTS in a population of 91 stroke subjects. Intra-rater agreement for elbow flexors and ankle plantar flexors were very good for R1, R2, R2-R1, and MTS scores.

Our study reports higher intra-rater reliability for all components of MTS than other studies reported in past. Mackey *et al*.[20] reported poor intraobserver reliability for MTS in biceps spasticity in children. This author used 3D Kinematics and not goniometer while author quoted various limitations of this method. Author stated use of 3D analyzer itself as one of the limitation. Children were seated rather than lying down for the ease of using 3D movement analyzer. Changes in marker placement are also quoted as limitation. We used universal goniometer in our study as this tool is easy to use, cost effective, and widely available in all kinds of settings. In another study, Boyd *et al*.[18] reported good intra-rater reliability for MTS in CP children. Fosang *et al*.[19] reported ICC range of various muscles in lower limb as Hamstrings, 0.68 to 0.90; gastrocnemius, 0.38 to 0.90; hip adductors, 0.61 to 0.93. There is wide variability in range but upper limit shows very good agreement. The author has cited many reasons for the variability. This study was conducted in CP children in whom change in tone was profound. Also, children were learning and had anticipation of stretch during the sessions. Higher reliability in our study could be attributed to some factors. This study has included large sample size and adult subjects with stroke. Adult subjects may not have anticipation and learning as it was in children. This study was performed in two sessions that may not be enough to cause learning among the subjects. Adult stroke subjects of 57.5 ± 13.5 days duration included in this study may have less fluctuation of tone. This finding raises scope for further evaluation of MTS among children and adult population as well as nature of spasticity and pattern of tone fluctuation in different conditions.

Test-retest reliability reported by Mehrholz *et al*.[22] for MTS in comparative study of MTS and MAS was moderate to very good (κ = 0.52 – 0.87) in severe brain injury adult subjects. In a previous study,[23] author has reported lesser inter-rater reliability in a population of 30 stroke subjects. Though this study was inter-rater reliability, it needs some elucidation because authors have concluded some interesting limitations as lack of experience and training in use of MTS as one of the factor for achieving low value. Our study was not confounded by this factor as rater was well experienced in handling stroke subjects as well as well trained for MTS. Study had begun only after approval of experienced Neuro-physiotherapists (experience of >10 years) for satisfactory use of MTS by rater in stroke subjects. Subjects included in our study were in range of 32 to 78 days after onset of stroke, whereas previous studies have large range of duration of stroke subjects. There are few limitations of this study. The interval between two sessions was two days and this can be one of the reasons to achieve higher intra-rater reliability in this study. One limitation can be the achievement of relaxed state in the subjects across the sessions by raters. Goniometer was attached in each session, although this was tried to overcome by use of standard landmark. One of the major limitations of this study can be the interval between assessments, which was two days. A study can be conducted with larger interval of one to two weeks.

## Conclusions

Authors conclude that intra-rater reliability of MTS in adult subjects with stroke is very good for elbow flexors and ankle plantar flexors. This tool is easy to administer and quantify spasticity. Future research should be directed to use this tool in various other conditions where spasticity is common, with pattern of change in tone fluctuation.

## References

[1] Sommerfeld DK, Eek EU, Svensson AK, Holmqvist LW, von Arbin MH (2004). Spasticity after stroke: Its occurrence and association with motor impairments and activity limitations. Stroke.

[2] Rathore SS, Hinn AR, Cooper LS, Tyroler HA, Rosamond WD (2002). Characterization of acute stroke signs and symptoms: Findings from the atherosclerosis risk in communities study. Stroke.

[3] Gray CS, French JM, Bates D, Cartlidge NE, James OF, Venables G (1990). Motor recovery following stroke. Age Ageing.

[4] Twitchtell TE (1951). The restoration of motor function following hemiplegia in man. Brain.

[5] Lance JW, Lance JW, Feldman RG, Koella WP (1980). Pathophysiology of spasticity and clinical experience with baclofen. Spasticity: Disordered motor control.

[6] Vattanasilp W, Ada L (1999). The relationship between clinical and laboratory measures of spasticity. Aust J Physiother.

[7] Haugh AB, Pandyan AD, Johnson GR (2006). A systematic review of the Tardieu Scale for the measurement of spasticity. Disab Rehabil.

[8] Pandyan AD, Johnson GR, Price CI, Curless RH, Barnes MP, Rodgers H (1999). A review of the properties and limitations of Ashworth and Modified Ashworth Scales as measures of spasticity. Clin Rehabil.

[9] Patrick E, Ada L (2006). The Tardieu Scale differentiates contracture from spasticity whereas the Ashworth Scale is confounded by it. Clin Rehabil.

[10] Vattanasilp W, Ada L, Crosbie J (2000). Contribution of thixotropy, spasticity, and contracture to ankle stiffness after stroke. J Neurol Neurosurg Psychiatr.

[11] Boyd RN, Ada L, Barnes MP, Johnson GR (2001). Physiotherapy management of spasticity. Upper motor neurone syndrome and spasticity.

[12] Pandyan AD, Gregoric M, Barnes MP, Wood D, van Wijck F, Burridge J (2005). Spasticity: Clinical perceptions, neurological realities and meaningful measurement. Disab Rehabil.

[13] Barnes MP, Barnes MP, Johnson GR (2001). An overview of the clinical management of spasticity. Upper motor neurone syndrome and spasticity.

[14] Tardieu G, Shentoub S, Delarue R (1954). In search of a technique for measuring spasticity. Revue Neurolique.

[15] Held J, Peierrot-Deseilligny E (1999). Reeducation motrice des avections neurologiques.

[16] Boyd R, Graham H (1999). Objective measurement of clinical findings in the use of botulinum toxin type A for the management of children with cerebral palsy. Eur J Neurol.

[17] Love SC, Valentine JP, Blair EM, Price CJ, Cole JH, Chauvel PJ (2001). The effect of botulinum toxin type A on the functional ability of the child with spastic hemiplegia a randomized controlled trial. J Neurol.

[18] Boyd RN, Barwood SA, Ballieu CE, Graham HK (1998). Validity of a clinical measure of spasticity in children with cerebral palsy in a double-blind randomized controlled clinical trial. Dev Med Child Neurol.

[19] Fosang AL, Galea MP, McCoy AT, Reddihough DS, Story I (2003). Measures of muscle and joint performance in the lower limb of children with cerebral palsy. Dev Med Child Neurol.

[20] Mackey AH, Walt SE, Lobb G, Stott NS (2004). Intraobserver reliability of the modified Tardieu scale in the upper limb of children with hemiplegia. Dev Med Child Neurol.

[21] Yam WK, Leung MS (2006). Interrater reliability of modified ashworth scale and modified tardieu scale in children with spastic cerebral palsy. J Child Neurol.

[22] Mehrholz J, Wagner K, Meissner D, Grundmann K, Zange C, Koch R (2005). Reliability of the Modified Tardieu Scale and the Modified Ashworth Scale in adult patients with severe brain injury: A comparison study. Clin Rehabil.

[23] Ansari NN, Naghdi S, Hasson S, Azarsa MH, Azarnia S (2008). The Modified Tardieu Scale for the measurement of elbow flexorspasticity in adult patients with hemiplegia. Brain Injury.

[24] Norkin CC, White DJ (1995). Measurement of joint motion, a guide to goniometry.

[25] Portney LG, Watkins MP (1993). Foundations of clinical research.

